# Dome-Type: A Distinctive Variant of Colonic Adenocarcinoma

**DOI:** 10.1155/2012/284064

**Published:** 2012-11-19

**Authors:** Giacomo Puppa, Mariella Molaro

**Affiliations:** ^1^Service de Pathologie Clinique, Hôpitaux Universitaires de Genève, 1211 Geneva, Switzerland; ^2^Division of Gastroenterology, “G. Fracastoro” City Hospital, Verona, Italy

## Abstract

*Introduction*. Ten cases of dome-type adenocarcinoma of the colon have been reported so far. Most of them were presented as early lesions, with endoscopic and microscopic distinguishing features. *Methods and Results*. A raised plaque was removed from the right colon during colonoscopy in a 56-year-old man. Histopathological examination showed a cancerized adenoma invading the submucosa with several typical features of dome-type adenocarcinoma, in particular the associated prominent lymphoid tissue. Immunohistochemistry showed retention of the mismatch repair proteins MLH-1, MSH-2, MLH-6, and PMS-2. *Conclusion*. We report an additional case of dome-type adenocarcinoma of the colon as an early, low-risk, and microsatellite stable tumor, indicating that this particular histotype may deserve specific consideration for both classification and management.

## 1. Introduction

Since the initial reports back in late 90's & 2000's by De Petris et al. [1] and Jass et al. [2] 10 cases of dome-type (DC) adenocarcinoma of the colon have been reported [1–7]. DC is considered a rare variant of carcinoma of the colon presenting as a nonpolypoid plaque lesion, it is thought to derive from the specialized columnar M-cells of dome epithelium, which makes up in association with the gut-associated lymphoid tissue the domelike masses that bulge into the gut lumen [2].

Originating from this specific microenvironment, the most important morphological feature of DC is the association with a prominent lymphoid stroma.

We would like to add an additional case of this particular histotype that was recently diagnosed in our institute.

## 2. Case Presentation

A 56-years male with amyotrophic lateral sclerosis was colonoscoped because of painful constipation. There was no case of colorectal cancer in his family history.

**Figure 1:**
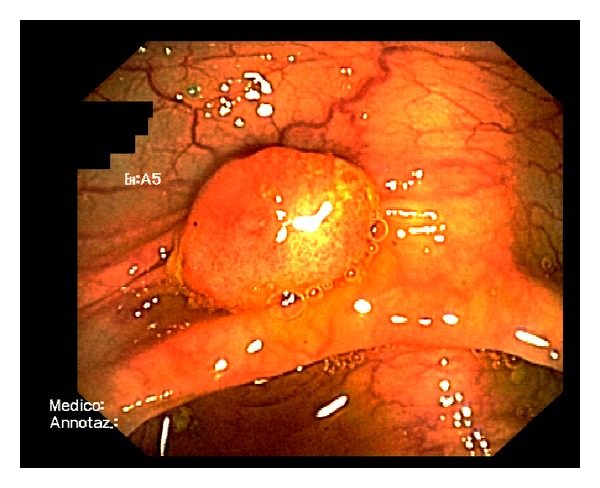
Conventional endoscopic image showing the dome-like lesion. A reddish rough mucosa can be seen in the top surface.

An 8-mm raised plaque was seen in the right flexure (Figure 1) and removed.

**Figure 2:**
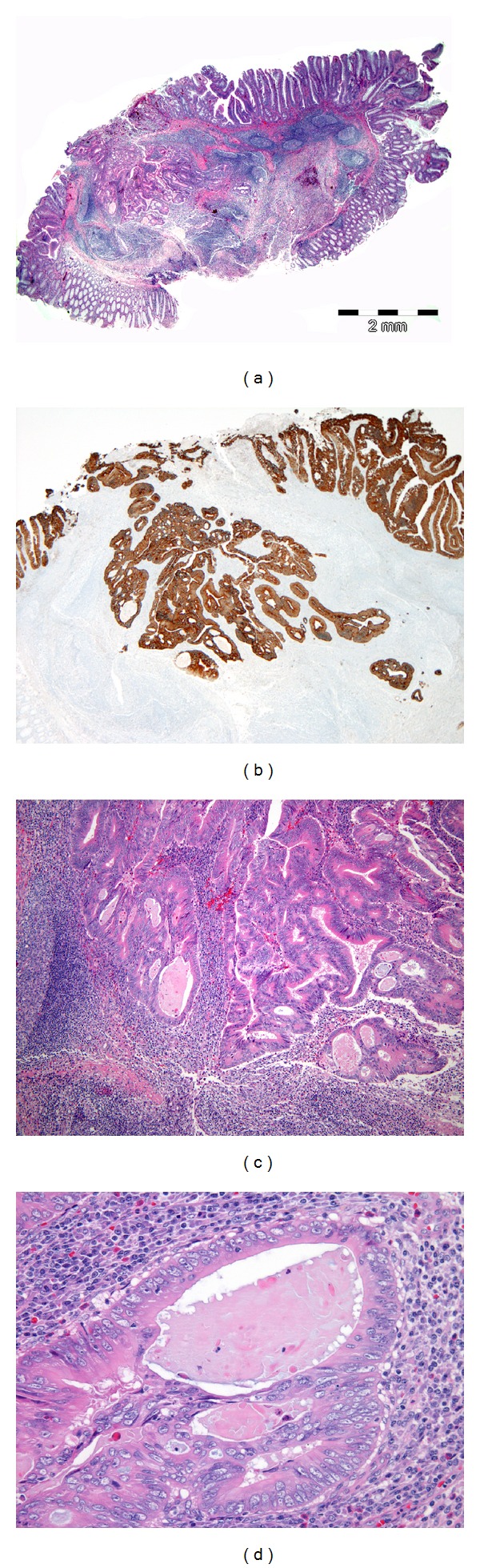
(a) Panoramic view of the tumor described. A well-demarcated tumor grows into the submucosa (hematoxylin-eosin, ×10). Overlying mucosa shows adenoma with high-grade dysplasia. Invasive adenocarcinoma associated with prominent lymphoid tissue encompassing several reactive germinal centres is observed in the submucosal layer. (b) Pan-Cytokeratin highlight tumor invasion. From the advancing edge few scattered foci of tumor budding arise (Cytocheratin AE1-3 ×20). (c) Histological architecture encompassing cystically dilated glands, in part cribriform arranged (hematoxylin-eosin, ×50). (d) The pink eosinophilic material filling the more dilated glands; a clear space separates the glandular epithelium from the intraglandular material (hematoxylin-eosin, ×200).

Routine histopathological examination showed a cancerized adenoma invading the submucosa associated with expanded lymphoid tissue encompassing several reactive germinal centres (Figures 2(a), 2(b), and 2(c)).

The cancer, arisen in a flat adenoma with high-grade dysplasia, was ulcerated superficially and the advancing edge appeared quite well circumscribed except for a more submucosa-invading tongue of neoplastic glands where some low-grade budding was observed (Figures 2(a), 2(b) and 2(c)).

The glands were in part cystically dilated containing a pink eosinophilic material, in part cribriform arranged (Figures 2(c) and 2(d)).

A clear space often separates the glandular epithelium from the intraglandular material.

Neoplastic cells lining the glands were columnar, single-layered, well-differentiated, eosinophilic (Figure 2(d)).

 No tumor infiltrating lymphocytes nor goblet cells were observed. Necrosis and desmoplasia also were absent.

The T1 adenocarcinoma was considered a low-risk lesion because of the absence of lymphovascular invasion, the low-differentiation grade, and the negative resection margin, therefore no hemicolectomy was performed.

**Figure 3:**
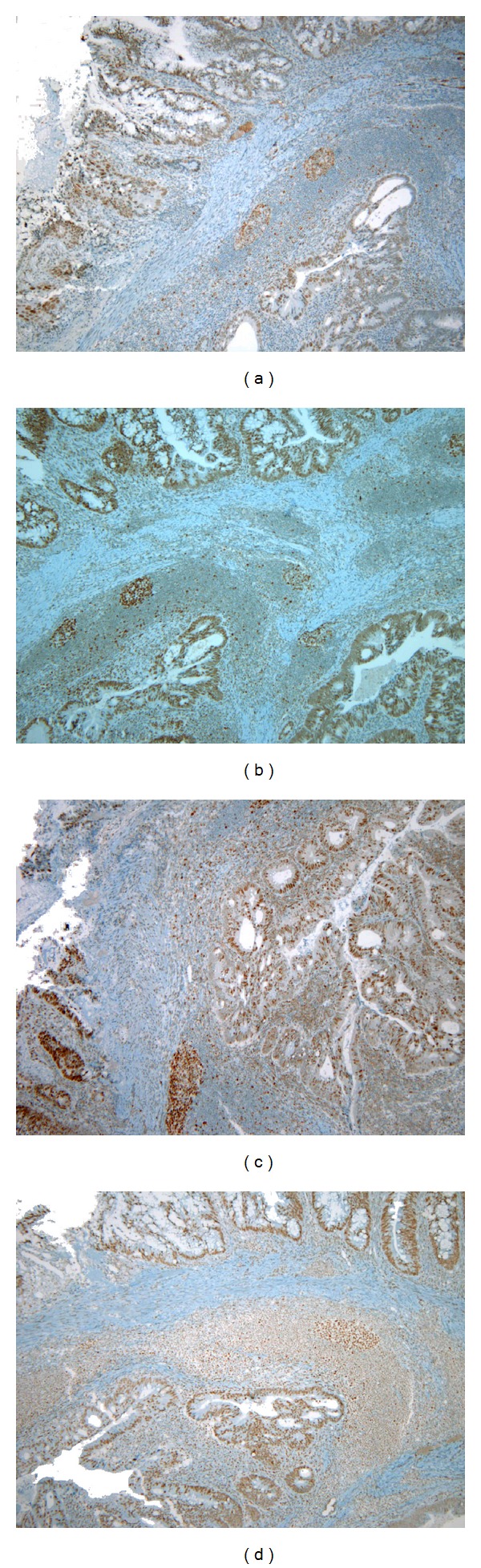
Immunohistochemical analysis of MLH-1, MSH-2, MSH-6, and PMS-2 protein expression in the DC. The neoplastic cells and the internal control (lymphocytes) are positive (a) for MLH-1 (×40), (b) for MSH-2 (×40), (c) for MSH-6 (×40), and (d) for PMS-2 (×40).

Immunohistochemistry showed retained expression of the mismatch repair proteins MLH-1, MSH-2 MSH-6, and PMS-2 in the neoplastic cells as well in the internal control (Figures 3(a), 3(b), 3(c), and 3(d), resp.).

The patient underwent a follow-up colonoscopy one year later: the mucosal biopsies from the polypectomy site showed at histology mild fibroinflammatory changes.

## 3. Discussion

DC may develop as sporadic-type colon cancer or in association with ulcerative colitis, [6] familial adenomatous polyposis, [2] hereditary nonpolyposis colorectal cancer, [1] and other positive family history of colorectal cancer, [2, 5] in both right and left colon, therefore DC is not associated to any specific mechanisms of tumour predisposition.

Some distinguishing features both macroscopic and microscopic are constantly present: the nonpolypoid appearance, the cell architecture, the cytology, and the presence of prominent lymphoid tissue; other features such as the tumor infiltrating lymphocytes, the intra-acinar necrosis, the remnants of a preexisting adenoma and foci of usual-type adenocarcinoma of the colon may be present or absent.

Most cases are reported in the early growth phase: eight over ten case reported are T1N0, [1–6] one is T2N0 [3], and the last one is T3N0 [7]. No recurrence is documented so far and the patient presented with this report is one-year recurrence-free.

The case reported here of DC is another early and low-grade lesion, lacking features of biological aggressiveness, microsatellite stable tumor, suggesting that this particular histotype may deserve a space in the classification of tumors of the colon and rectum.

In the management of neoplastic colonic polyps DC *per se* may identify a low-risk malignant lesion, influencing the treatment decision-making process.

## References

[1] De Petris G, Lev R, Quirk DM, Ferbend PR, Butmarc JR, Elenitoba-Johnson K (1999). Lymphoepithelioma-like carcinoma of the colon in a patient with hereditary nonpolyposis colorectal cancer. *Archives of Pathology and Laboratory Medicine*.

[2] Jass JR, Constable L, Sutherland R (2000). Adenocarcinoma of colon differentiating as dome epithelium of gut-associated lymphoid tissue. *Histopathology*.

[3] Asmussen L, Pachler J, Holck S (2008). Colorectal carcinoma with dome-like phenotype: an under-recognised subset of colorectal carcinoma?. *Journal of Clinical Pathology*.

[4] Clouston AD, Clouston DR, Jass JR (2000). Adenocarcinoma of colon differentiating as dome epithelium of gut-associated lymphoid tissue. *Histopathology*.

[5] Coyne JD (2012). Dome-type colorectal carcinoma: a case report and review of the literature. *Colorectal Disease*.

[6] Stewart CJR, Hillery S, Newman N, Platell C, Ryan G (2008). Dome-type carcinoma of the colon. *Histopathology*.

[7] Yamada M, Sekine S, Matsuda T (2012). Dome-type carcinoma of the colon, a rare variant of adenocarcinoma resembling a submucosal tumor: a case report. *BMC Gastroenterology*.

